# Use of Artificial Intelligence Tools for Research by Medical Students: A Narrative Review

**DOI:** 10.7759/cureus.55367

**Published:** 2024-03-01

**Authors:** Karanvir Singh Jhajj, Pooja Jindal, Kirandeep Kaur

**Affiliations:** 1 Medicine and Surgery, Dayanand Medical College and Hospital, Ludhiana, IND; 2 Pharmacology and Therapeutics, Dayanand Medical College and Hospital, Ludhiana, IND

**Keywords:** artificial intelligence in scientific writing, medical writing, medical student, medical research methods, medical artificial intelligence

## Abstract

With the advent of the era of digitalization, a new door has opened for the development of artificial intelligence (AI)-driven tools/algorithms that can help analyse the huge amount of data uploaded onto the cloud. AI-based tools/algorithms have created a niche in the field of research. AI has enabled researchers and practitioners to access and evaluate an enormous number of scientific papers more effectively. This can link similar studies from the past and highlight research gaps, thus accelerating the literature review, evidence generation, and knowledge discovery process. Medical students can obtain help from various AI-based solutions for literature organization and citations. These tools/algorithms facilitate secure information exchange, collaborative research efforts, and communication among multiple research centres. However, AI-driven research requires the guidance and supervision of human experts for better accuracy, coherence, and credibility of the content entering scientific databases. The key objective of this review is to discuss and evaluate various AI-based tools/algorithms and their key features that can assist medical students in medical research.

## Introduction and background

Although there have been several attempts to define artificial intelligence (AI), John McCarthy defined it as the science and engineering of making intelligent machines, especially intelligent computer programs. It is related to the similar task of using computers to understand human intelligence, but AI does not have to confine itself to biologically observable methods [1]. 

With ChatGPT penetrating every sphere of social and professional life, there has been an increasing hype surrounding the application of AI tools/algorithms to more complex challenges in healthcare, such as enabling diagnostics and facilitating drug discovery and medical research [2]. AI is not a single technology but a constellation of machine learning (ML), neural networks, and natural language processing (NLP). ML is a branch of AI that focuses on developing algorithms that imitate the way humans learn, gradually improving their performance and accuracy based on the data they ingest [3]. Artificial neural networks are simulated neurons and are a biologically derived subset of AI that are loosely modeled to mimic the structure and function of the human brain. NLP is a discipline that lies at the junction of computer science, linguistics, and ML and is associated with teaching computers to interpret, manipulate, and comprehend the nuances of human language. 

However, even in the 21st century, there remains a conservative school of thought against the use of AI by medical student researchers and the research community at large. There is an ongoing debate on whether AI is replacing the creativity of young researchers with machine-generated language, providing an easy way out, or is only being used to help guide research conducted by them. As expressed by Rind, student researchers face many problems and challenges including topic selection, lack of access to appropriate literature, lack of research knowledge and skills, poor reference management, and data collection and interpretation [4]. While AI tools/algorithms can help mitigate a lot of these issues for students, there remain certain obstacles including privacy and security concerns, a lack of human engagement, and technological and data accuracy limits [5]. 

Various AI applications have been developed in the last few years, which can help seasoned researchers streamline the processes of literature review, identifying research questions, evidence synthesis, and manuscript and grant writing [6]. Researchers can also work remotely on a shared platform while being separated by continent and sea. Our goal is to provide an overview of the existing tools available on the Web for young scientists and research novices that can help boost their entry into the ginormous field of research.

## Review

Methods

Search Strategy

We conducted a thorough search on the internet to explore various AI-driven tools/algorithms that could have significant potential in medical research. Utilising the major search engines we searched for “AI tools/algorithms for *(a)* for research”, where* (a)* stood for the major stages in conducting research. These included: "Literature search", "Literature mapping”, "Reference management”, "Writing and finalisation” and “Collaboration”. Our internet search period ended in November, 2023.

Selection Criteria

Tools/algorithms that provided a free trial period were preferred. Further, among the tools requiring a subscription, those catering to individual researchers and those that could be bought online were preferred. We compared the various features of these tools and prepared a comprehensive list of these tools and their distinguishing features. The major tools/algorithms in certain sections have been compared and contrasts have been provided in tabulated forms.

Results

AI tools/algorithms have been divided into the following broad categories for medical research: literature searches, literature mapping, reference managers, tools for writing and finalisation and collaboration.

 Literature Search Tools 

Several AI-driven search platforms have been developed in the AI revolution to methodically sieve through various sources of scientific literature and efficiently retrieve and discover data, ensuring that no relevant literature is overlooked. With the modeling of AI tools/algorithms that can understand the context in which words are used, can lead to more accurate and relevant results [7]. Specific features, such as author name disambiguation, query manipulation, and automatic indexing of articles are useful in providing succinct summaries of relevant studies to facilitate the design and storage of papers of interest. This helps to alleviate the burden of the initial stages of the literature review.

Out of the various multidisciplinary databases employing AI algorithms, three databases have been discussed below along with their unique features.

PubMed (https://pubmed.ncbi.nlm.nih.gov/ ): It is an open-access, go-to resource supporting a database of more than 36 million citations and abstracts of biomedical literature. MEDLINE (Medical Literature Analysis and Retrieval System Online), the largest subset of PubMed, comprises of citations from selected journals, articles indexed with MeSH (Medical Subject Headings), and other curated metadata. Recently, PubMed has begun to use AI to enhance the sorting of search results using the "Best Match" option. Further, it analyses user behavior to suggest similar articles of interest [8].

Scopus (https://www.elsevier.com/en-in/solutions/scopus): Concerning books, scientific journals, and conference proceedings that are subject to peer review, Scopus is Elsevier’s greatest multidisciplinary abstract and citation database that employs AI to enhance search, citation analysis, and research evaluation. It also provides a free feature for non-subscribed users called Scopus Preview, allowing knowledge about Scopus content coverage and source metrics.

Embase (https://www.elsevier.com/en-in/solutions/embase-biomedical-research): It is a highly efficient database in which we can conduct systematic literature reviews using a tailored PICO (population, intervention, control, and outcomes) method in a search. It provides access to the relevant information from trusted sources. It has full indexing with the Emtree thesaurus. It is a paid and expensive database that is unavailable in many institutions. 

These three databases have been compared in Table 1.

**Table 1:** Comparison between PubMed, Scopus, and Embase based on their discipline, coverage, updating, and accessibility.

Characteristics	PubMed	Scopus	Embase
Discipline	Medicine or biological sciences	Multidisciplinary	Broad biomedical scope with in-depth coverage of pharmacology and clinical research
Coverage	Dates back to 1966 (selectively to 1809) Over 36 million records in over 5293 indexed journals	2.4+ billion cited references dating back to 1970 (selective records from 1788 onwards). 94+ million records with over 25,000 indexed journals	Date back to 1947 and selectively to 1902. Contains over 44.7 million records with an average of 8000 new entries daily in 3293 journals unique to Embase and 8451 currently published journals
Updating	Daily	Daily	Daily
Accessibility	Free	Requires paid subscription	Amongst the most expensive databases

The rest of the search engines are described below.

Semantic Scholar (https://www.semanticscholar.org/): It offers extremely succinct summaries of a paper's primary goals and findings, which are produced utilizing expert background knowledge and NLP approaches. All the papers that pique the researcher's interest are arranged and stored in their virtual library, which they may access through their account. This facilitates the easy exchange of documents across colleagues. This suggests relevant related papers based on an AI-powered engine based on prior selections. 

Sourcely (https://www.sourcely.net/): It is an AI-powered tool for finding, summarizing, and formatting sources for academic papers. Researchers can even apply advanced filters to tailor our search by publication year, authorship, relevance, etc.

SciSpace (https://scispace.com/): It provides summaries and conclusions of individual articles in an easy-to-comprehend language, bypassing the complex scientific jargon. SciSpace Copilot is a Chrome extension that can instantly answer questions and break down concepts for the ease of the user while perusing research papers.

System Pro (https://about.system.com/product/system-pro): System Pro aids in finding, synthesizing, and contextualizing literature, surfaces new insights, and enhances the research experience. The search provides an accurate and up-to-date overview of the relevant research that allows the researcher to quickly pinpoint where there is agreement and disagreement. It provides the entire range of topics statistically and mechanistically related to the search, supported by precise data and citations. 

SciLynk (https://www.scilynk.com/): Scilynk is an AI search engine to find and organize research papers more efficiently. Researchers have the choice to filter the results based on articles and book chapters. The research field can be further filtered by the authors, open access, and concepts. Researchers can narrow down the field by using the name of a journal in the keywords. This cutting-edge tool allows the creation of collections to save research papers for future perusal. This allows seamless integration with the preferred reference manager. Lynx AI is a personal research assistant within this tool that can summarize search results and extract important data.

Scinapse (https://www.scinapse.io/): It is an AI-powered research intelligence engine. By gathering and evaluating academic papers rather than just offering a search and listing service, Scinapse provides useful insights that address the problems of an abundance of research papers, gaps in the literature, coming up with new ideas, and staying current with emerging trends. From each document, the program retrieves useful information, such as relevant topics and patent data, and provides a more comprehensive research experience through its database. 

Perplexity (https://www.perplexity.ai/): It is a robust search and answer engine that has been specifically developed to answer complex and intricate questions. This allows for the generation of content and paraphrasing of text that is more scientifically acceptable. It can be used to learn about the research field and to find the latest articles within the scope of the topic. It has a Pro upgrade that can be used for Copilot, ChatGPT-4, etc. 

R discovery (https://discovery.researcher.life/): It is a mobile app that expedites the research discovery process by providing the most recent and relevant content in the field of interest. It offers an audio rendition of the article’s abstract and its translation into 27 languages. The user can store and sync documents to the integrated reference manager and share them with others.

Scite.ai (https://scite.ai/): Scite, a Brooklyn-based organization helps academics better discover and understand research articles through Smart Citations, citations that highlight the context of the citation and describe whether the article provides supporting or conflicting information. It assists scholars in locating appropriate sources for literature reviews, filtering references based on relevance, date, citations, and discipline, and enables easy access to full-text publications. Its free resources, especially the Zotero plugin and browser extension, allow users to further ease the literature evaluation process.

Literature Mapping 

Literature mapping software are instrumental in discovering scholarly articles by exploring connections between publications. They enable researchers to generate visual maps of the existing literature based on citations, authors, funders, keywords, and other metadata [9]. They aid in providing a holistic understanding of the research domain and expose research gaps of significance. This is a very effective way of visualizing a research database related to a topic in a more tangible manner.

**Figure 1:**
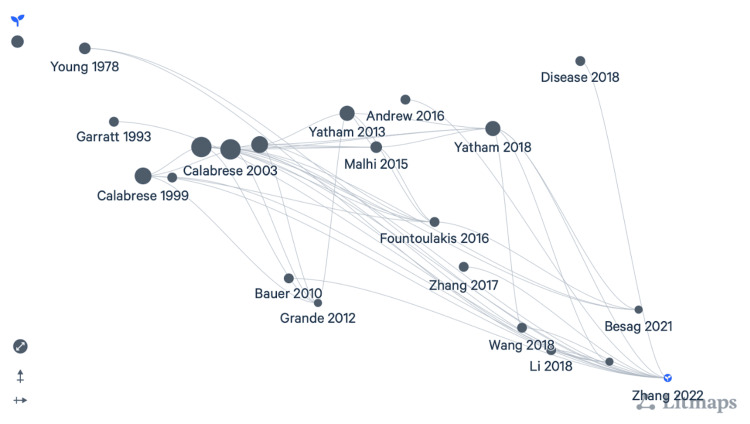
A Litmap that has been generated after choosing a seed paper from suggestions, using keyword search “Lamotrigine and mood disorders”

Litmaps (https://www.litmaps.com/): With its advanced discovery tools, Litmaps helps to filter results by date range, author, and keyword and also allows access to second-order citations from a discovery graph. It generates a visual map that exhibits the relevance and temporal relationships between the selected articles. Unlike other tools, it uses a citation network to present relevant recommendations. Litmaps can be shared easily on Twitter (Now X, X Corp., San Francisco, California, United States) and Facebook (Meta Platforms, Inc., Menlo Park, California, United States) using a link and can be exported as an image for inclusion in research. Litmaps Pro notifies about new additions and developments in the selected field of research. For reference, look at Figure 1.

Research Rabbit (https://www.researchrabbit.ai/): Dubbed "Spotify for papers", it provides AI-based intuitive exploration and personalized recommendations, generating powerful interactive visualizations. This allows author discovery based on the selected research papers. It allows for collaboration on collections and provides an opportunity to comment. Zotero integration is another gem that allows for syncing of the researcher's reference library and search tool for a more efficient and time-saving experience. 

Connected Papers (https://www.connectedpapers.com/): Connected Papers generate a graph after analyzing a large quantum of papers based on co-citation and bibliography coupling, using the Semantic Scholar database. Similar papers are clustered together, whereas less similar papers are placed farther away.

Inciteful (https://inciteful.xyz/): Inciteful differs from other software by emphasizing the underlying structure that citations provide. This helps create a citation network centered around the paper(s) of choice. It analyses this network to surface the most recent, interesting, and state-of-the-art data. Using citations, the Paper Discovery tool creates a network of publications, analyzes the network using network analysis methods, and provides the user with the knowledge needed to quickly become up-to-date on that issue. Another tool called the Literature Connector allows the user to enter two papers and provides interactive visualization that demonstrates how the literature connects them. It is designed for interdisciplinary scholars attempting to bridge these two areas.

Lateral (https://www.lateral.io/): An AI-driven tool that provides a single free trial followed by paid access. One can upload all PDFs, search the database, and add papers. It then analyzes all of them together to produce concepts and highlight keywords for faster reading. It draws connections between the papers. All relevant data are exported into a table that makes the literature review process much quicker.

Reference Management

AI-driven reference management tools/algorithms are vital for efficiently organizing, citing, and retrieving relevant literature. It allows researchers to create a bibliographic database that can be retrieved and accessed at any stage of research. References can easily be formatted into journal-specific requirements. AI tools/algorithms can help find the correct sources, identify the correct citation style, and ensure that citations are correct and consistent. Special algorithms for systematic reviews and facilitating the sharing of reference libraries among researchers allow for collaboration and shared decision-making concerning paper inclusion and screening.

Zotero (https://www.zotero.org/): Zotero is a free tool that allows researchers to collect, organize, annotate, cite, and share research. One can directly save articles from a browser using the Zotero extension. It facilitates organization by sorting items into collections using keywords. It can instantly create references and bibliographies directly inside MS Word (Microsoft Corporation, Redmond, Washington, United States). A Zotero library can be shared free of cost. It can also be accessed as and when required.

Humata (https://www.humata.ai/): Humata is an innovative question-and-answer (Q&A) AI platform that helps summarize findings, provides prompt responses to queries regarding data and files, and lets users compare various documents. It provides answers together with citations so that users can trace the source of their insights. This allows the user to include its PDF on any webpage with a single click. The Q&A system makes it easy for users to navigate through complex and extensive research without spending hours reading and analyzing the data. 

**Figure 2:**
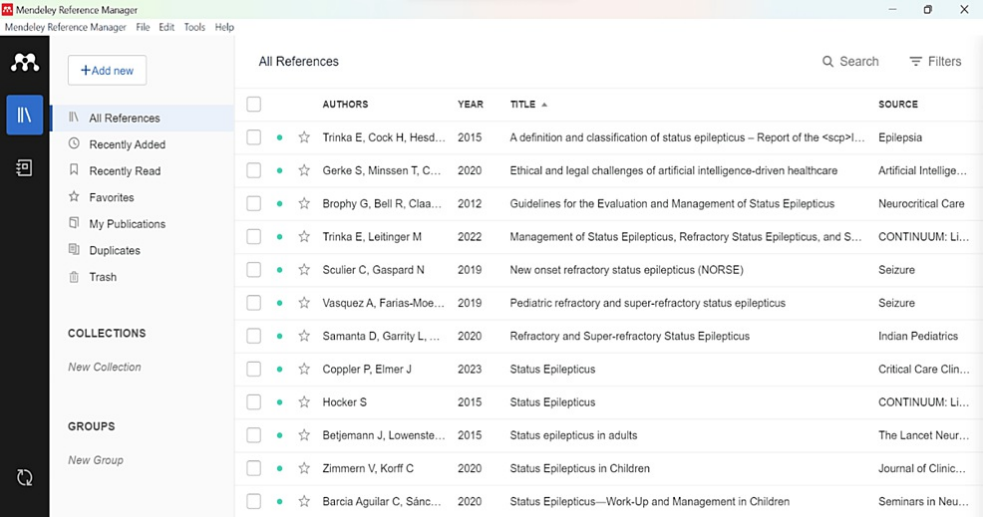
A Mendeley library generated after adding research articles from PubMed.

Mendeley (https://www.mendeley.com/): Mendeley is a free AI-driven reference manager that facilitates the user to save, arrange, note, share, and cite references and study information. It can automatically generate bibliographies and import papers from other research software. Its AI-powered recommendation engine and collaborative workspace encourage researchers to interact and collaborate easily with peers and other researchers online. This assists the researcher in finding relevant research articles based on what they are reading. With the help of Mendeley, researchers can access the papers online. It provides an opportunity to create a completely individualized database. One can create 10 databases for 10 projects (Figure 2).

Papersapp (https://www.papersapp.com/): It is an innovative AI tool assisting in the organization, discovery, connection, and writing of scientific literature. It automatically identifies and matches imported research papers with complete metadata, available supplemental data, full texts, and citations. This allows for the creation and assignment of tags, labels, and Watch folders to organize papers efficiently. It has a searchable database of nearly 130 million articles that can be accessed using its natural language processing-based discovery tool. It has features of real-time collaboration and cross-device syncing. It provides the researcher with tools to annotate and cite using SmartCite.

EndNote (https://endnlanguage=en): It is an AI-based reference management software with features to create a searchable personal library for all the references and reference-related materials. The EndNote Sync command synchronizes references between up to three computers, an EndNote Web library, and an iPad or iPhone. It allows the researcher to work smoothly with up to 400 co-authors and collaborators by establishing a sharing team and granting access to the synced library. The user can cite references in word processor documents. Even though it is widely recognized, EndNote costs much more than its competitors.

Paperpile (https://paperpile.com/): It’s built for Chrome and Google apps (Google LLC, Mountain View, California, United States) and provides a 30-day free trial. This allows the researcher to organize papers into folders, labels, and stars. Import data directly from Google Scholar, PubMed, ArXiv, and thousands of supported publisher sites. The Paperpile button is used to save a reference, PDF, or supplementary data file. Highlights and comments on PDFs. It’s integrated with Google Docs (Google LLC) for seamless reference management

Writing and Finalising 

Several AI-driven applications have come into the limelight as co-writers based on natural language processing. This accelerates the manuscript writing process while also providing grammar checks, syntax corrections, vocabulary suggestions, and precision editing tools. They are useful in enhancing the comprehensibility and readability of the text while helping the researcher navigate technical jargon and present their results in the best possible language.

Moonbeam (https://www.gomoonbeam.com/): Moonbeam is a long-form writing AI assistant. Its wizard tool can instantly transform messy notes into useful outlines and paragraphs. Luna is the AI behind Moonbeam, and with a few instructions, it can create a well-written first draft. It is equipped with the GPT-4 powered SmartChat for precision editing, effortless content creation, etc. It provides a Google Docs-like experience tailored specifically for AI-generated content.

AI Writer (https://ai-writer.com/): AI Writer is a sophisticated AI text generator that has the unique feature of citing sources for everything it writes. These citations can be manually verified. With the help of its search engine optimization (SEO) editor, this effective software first analyzes the search engine results to learn what Google is looking for and then provides users with the right keywords. Working as a (Sub)Topic Discoverer, it assists users in finding the right subject for their subsequent article. The Text Rewording function allows scholars to republish a specific piece of content. 

Jenni.ai (https://jenni.ai/): An AI-based tool for writing, citing, and editing has been developed, especially for scientific writing. The AI Autocomplete feature allows for bypassing the writer’s block. Jenni allows the researcher to upload the PDFs of related research and base recommendations on the latest research and those PDFs along with in-text citations. Furthermore, it can summarize PDFs and answer questions with its AI chat assistant. It also allows for the creation of a research library for easy citations. It has a built-in plagiarism checker that allows the researcher to check the final document before exporting.

Trinka.ai (https://www.trinka.ai/): Trinka AI, powered by Enago (New Jersey, United States), is a next-generation paraphraser, grammar checker, and custom-built language enhancement tool for academic and technical writing. Trinka polishes writing tone, delivery, and phrasing to conform to academic conventions. The most advanced text similarity detection algorithm, iThenticate, and the largest paid publication database covering all scientific fields provide the highest quality plagiarism check. It can proofread and edit an MS Word document and assess the manuscript for 20+ checkpoints corresponding to journal editorial checks. Citation Checker helps in citing strong and credible citations. It can compare the ideas in a paper with millions of publications and publication trends to find an appropriate journal. Trinka personalized the writing experience by enabling users to create their dictionaries.

Writefull (https://www.writefull.com/): Developed by a team of PhDs in AI and linguistics, Writefull is a software that has been specifically designed for research writing. Writefull offers comprehensive and accurate language feedback that enhances the quality of the research paper. It provides a set of AI widgets, such as a Paraphraser, Abstract Generator, and Academizer, to assist academic writers and researchers. Writefull’s Title Generator generates titles based on the abstract. Furthermore, it delivers a collection of example sentences and a database of academic texts to help users refine their writing. 

Paperpal (https://paperpal.com/): A tool that enhances the success of scientific writing using real-time subject-specific language suggestions. It is a tailored tool for researchers and academic writing, with the accurate detection of complex grammar errors and rephrasing suggestions. Furthermore, it reviews the documents and checks for structural and technical inconsistencies. It looks for the completion of a manuscript using journal-specific submission requirements and ultimately reduces desk rejection based on simple techniques.

Penelope (https://www.penelope.ai/): It is an online tool that assesses the structure, declarations, statistics, and referencing of academic manuscripts written in MS Word. The software offers features that ensure that the manuscript satisfies journal requirements by checking for the ethics approval section and statement, including informed consent, presenting the abstract in a structured or unstructured manner, and other technicalities that can be customized per journal guidelines.

Grammarly (https://www.grammarly.com/): Grammarly is an AI-driven tool that has been extensively used in writing forums. It can also be used to write research papers. It provides real-time feedback that improves document quality during the writing process. It is a grammar-checker, plagiarism-checker, and a vocabulary improvement tool. It can quickly suggest full-sentence rewrites to make a clearer and concise point.

Quillbot (https://quillbot.com/): Originally designed as a paraphrasing tool, Quillbolt is now a free one-stop shop for all the various needs of manuscript writers. It includes a plethora of features, such as grammar checkers, plagiarism checkers, translators, citation generators, and summarizers. An AI-powered thesaurus is used to suggest a list of potential synonyms. It can be integrated directly into Chrome and MS Word. During paraphrasing, it can compare outcomes and choose from eight predefined modes that help improve the fluency and readability of the text. 

Table 2 gives a detailed comparison between four writing and finishing tools.

**Table 2:** Comparison between Paperpal, Quillbot, Grammarly, and Writefull based on their availability, usage, and pricing.

Features	Paperpal	Quillbot	Grammarly	Writefull
Availability	Paperpal web app and Microsoft Word	Web page and macOS	Windows, macOS, iOS, Android, and the Grammarly desktop and mobile app	Microsoft Word, Overleaf (a LaTeX editor), and the Writefull web app
Used for	Academic writing with a formal tone	Any writing task just like Grammarly. Not specific for academic writing	Any writing task including chatting, essay writing, etc. Not very useful in academic writing	Students or anyone working in academia looking for a formal writing assistant
Pricing	Limited free access. Unlimited features like grammar correction, plagiarism checker, unlimited copilot uses, consistency checks, submission readiness checks, etc. at $9.91/month.	The free account provides access to all services, just with limited capabilities. Users have three different paraphrasing modes, can summarise up to 5000 characters, and have access to the grammar checker for free. At $8.33/month, it is cheaper than Grammarly (but limits plagiarism checks to 20 pages). It has a special co-writer.	Features like grammar, spelling, and punctuation correction are available for free. The rest of the features require a premium subscription (approx. $12/month) including unlimited plagiarism checks.	It provides limited free access to paraphrasing, abstract generator, GPT detector, etc. Its premium subscription (~ $29/year) allows access to all of Writefull’s language suggestions, has a higher quota for the Sentence Palette, Language Search, Title Generator, TexGPT, etc.

Collaborations 

With the Internet boom, scientific transcontinental collaboration has increased exponentially. AI-powered tools/algorithms allow seamless collaboration among medical professionals, researchers, and institutions. This allows for complex data analysis and interdisciplinary collaboration. 

Bit.ai (https://bit.ai/): Bit.ai is a powerful all-in-one document management and collaboration application that allows users to create, share, and collaborate on documents in real time. It offers a variety of features, such as rich media embedding, customizable templates, visual web links, social media posts, and surveys that enable users to create documents, presentations, wikis, knowledge bases, and projects. With @mentions, real-time notifications, shared workspaces, permissions, and guest access, it allows small teams and organizations to connect and coordinate efficiently. Its built-in document-tracking feature provides users with real-time insights into their shared documents and helps study user engagement.

Labarchives (https://www.labarchives.com/): Dubbed the electronic lab notebook for modern scientists, it allows better data management, connectivity, and collaboration with cloud support. Data are stored on a secure and easily accessible platform. It is purpose-built for scientists and allows browsing of even DNA files to meet needs. Suited for teams of any size.

Discussion

Our study attempted to provide an overview of the major AI tools/algorithms that can be used easily by student researchers to launch their research careers and accelerate the research process. Various tools for literature search and review like PubMed, Semantic Scholar, etc. can assist with literature mapping such as Research Rabbit and Litmaps, and reference management tools such as Zotero to streamline the entire process. Further, tools like Paperpal, Jenni.ai, Labarchives, etc. can automate the editing and review process, allowing seamless collaboration between multiple reviewers [10].

Whether AI helps or replaces research is a matter of debate in nearly every academic circle and society. While AI tools can help generate long and apparently well-structured and researched drafts from a few pointers and make the process time-efficient and quick, is it acceptable at the cost of fabricated references and quotations [10-11]?

AI tools, such as ChatGPT, can generate plausible-sounding but erroneous answers. Several examples have provided references for studies that do not exist. A study conducted by Gao et al. in collaboration with the University of Chicago demonstrated that blinded human reviewers could correctly identify only 68% of AI-generated abstracts [12]. If only human reviewers were relied on, this could lead to seepage of AI-generated texts into the hallowed scriptures of scientific literature, which could prove disastrous. 

Therefore, the integrity of the scientific method must be preserved. Though machines play an important role, it has to be a human mind that thinks critically and suggests the hypothesis, designs the experiment, and finally, analyses the data [13]. The following ethical and legal issues must be considered before using AI in medical research and healthcare. Ethical issues include informed consent to utilize a patient’s data, safety and transparency, algorithmic fairness and biases, and data privacy. Legal issues like safety and effectiveness, liability, data protection and privacy, cybersecurity, and intellectual property law have gained significant scrutiny in the US and Europe [14].

Limitations

The main limitations that should be considered during the use of AI-powered research applications for conducting medical research according to many organizations, including the European Union Agency for Fundamental Rights (EUFRA), are given below.

Data and source quality: AI algorithms greatly depend on the caliber and applicability of the training data. Biases, errors, or missing information in the input data can affect how reliable the research findings are. Researchers need to make sure that the AI models' data sources are reliable, subject to peer review, and inclusive of the medical field.

Interpretability and explainability: Many AI models, especially deep learning models, operate as "black boxes". They provide predictions without clear explanations of how they arrived at those conclusions. In medical research, interpretability is crucial. Researchers need to understand why an AI model made a specific diagnosis or prediction to gain trust and confidence in its results.

Clinical context and human expertise: AI algorithms lack clinical judgment and context. They can provide insights based on patterns in data, but human expertise is essential for interpreting results and making informed decisions. Researchers should collaborate with healthcare professionals to validate AI findings and integrate them into clinical practice.

Ethical considerations: AI algorithms may inadvertently perpetuate biases present in historical data. For example, if a dataset primarily includes data from a specific demographic group, the AI model may not generalize well to other populations. Thus, researchers must be cautious about potential biases and ensure that ethical guidelines are followed during data collection and model training.

Data privacy and security: Handling sensitive patient data requires strict adherence to privacy regulations (such as the Health Insurance Portability and Accountability Act (HIPAA) in the United States). Thus, AI systems' role in protecting patient privacy and preventing unauthorized access to sensitive information must be ensured.

Generalization and validation: AI models trained on one dataset may not generalize well to new, unseen data. Researchers need to validate their models on diverse datasets to assess their performance across different scenarios. Overfitting (when a model performs well on training data but poorly on new data) is a common concern that needs careful validation.

Regulatory approval and adoption: Before implementing AI solutions in medical settings, regulatory bodies (such as the FDA) require rigorous evaluation and approval. Also, the adoption of AI tools in healthcare institutions involves challenges related to infrastructure, training, and acceptance by healthcare providers [15,16].

Advantages

Intelligence augmentation, in contrast to classic AI, allows the development of tools for augmenting and supporting human cognition. It does not take over the task entirely but rather complements human endeavors. It is a variant of artificial intelligence, but human-piloted, built for virtual, automated assistance, especially for mundane and repetitive tasks, clearing more time for creative ventures [17].

While there are several disadvantages to relying solely on AI, this should not discourage young and upcoming researchers and even seasoned professionals from taking full advantage of this upcoming technology. Although AI cannot be used to generate novel research ideas, it can give volume to ideas, provide shape to thoughts, and help create an initial draft. Several AI research assistants can aid researchers by finding academic papers, summarizing them, highlighting areas of uncertainty, and providing a crisp and quick understanding of the current research on the topic of interest. Providing insights into gaps in research that can be potential research ideas. Certain writing tools can even assist in suggesting the title and subheadings to make the paper more comprehensible [6]. AI tools/algorithms can mitigate the time-consuming processes of editing, formatting, and paraphrasing. Furthermore, tools such as Copyleaks (https://copyleaks.com), Turnitin (https://www.turnitin.com/), Originality (https://originality.ai/), and GPTZero (https://gptzero.me/) can be used to avoid desk rejection. Specialized tools for reference management and citations have significantly decreased researchers’ organizational workload. AI-bots for assistance, such as a CoPilot, can assist researchers in virtually every aspect of the process.

More complicated research endeavors, such as systematic review and meta-analysis, can only be aided and streamlined by AI-based tools, such as Rayyan [18] and Covidence (Melbourne, Victoria, Australia), but require the application of the human mind and comprehension. 

One should always remember that the scientific writing process, even if it may be AI-driven, requires guidance and supervision of human experts for better accuracy, coherence, and credibility of the content entering the literature [6]. As an eminent American writer, Elbert Hubbard has said, “One machine can perform the work of 50 ordinary men. No machine can do the work of one extraordinary man” [19]. It must be emphasized that the copilot can never become a pilot and that AI can never replace human intelligence but is only limited to assisting and augmenting the research experience.

## Conclusions

Various AI-powered tools have been introduced into the research atmosphere, all of which have great and novel potential to propel the pace of research. These can provide a new entry point and guide new researchers and act as an aid to seasoned researchers, looking to reduce their workload. With tools to markedly reduce the time spent on literature searches, reference management, and even assisting in writing and finalization, these surely will leave an impact on scientific research. While these tools have been getting hype for a while, cautious use is recommended to maintain the sanctity of scientific literature.
